# Use of advanced neuroimaging and artificial intelligence in meningiomas

**DOI:** 10.1111/bpa.13015

**Published:** 2022-02-25

**Authors:** Norbert Galldiks, Frank Angenstein, Jan‐Michael Werner, Elena K. Bauer, Robin Gutsche, Gereon R. Fink, Karl‐Josef Langen, Philipp Lohmann

**Affiliations:** ^1^ Department of Neurology Faculty of Medicine University Hospital Cologne University of Cologne Cologne Germany; ^2^ Institute of Neuroscience and Medicine (INM‐3, ‐4) Research Center Juelich Juelich Germany; ^3^ Center for Integrated Oncology (CIO) Universities of Aachen Cologne Germany; ^4^ Functional Neuroimaging Group Deutsches Zentrum für Neurodegenerative Erkrankungen (DZNE) Magdeburg Germany; ^5^ Leibniz Institute for Neurobiology (LIN) Magdeburg Germany; ^6^ Medical Faculty Otto von Guericke University Magdeburg Germany; ^7^ Department of Nuclear Medicine University Hospital Aachen Aachen Germany; ^8^ Department of Stereotaxy and Functional Neurosurgery Faculty of Medicine University Hospital Cologne University of Cologne Cologne Germany

**Keywords:** MRI, PET, radiogenomics, radiomics

## Abstract

Anatomical cross‐sectional imaging methods such as contrast‐enhanced MRI and CT are the standard for the delineation, treatment planning, and follow‐up of patients with meningioma. Besides, advanced neuroimaging is increasingly used to non‐invasively provide detailed insights into the molecular and metabolic features of meningiomas. These techniques are usually based on MRI, e.g., perfusion‐weighted imaging, diffusion‐weighted imaging, MR spectroscopy, and positron emission tomography. Furthermore, artificial intelligence methods such as radiomics offer the potential to extract quantitative imaging features from routinely acquired anatomical MRI and CT scans and advanced imaging techniques. This allows the linking of imaging phenotypes to meningioma characteristics, e.g., the molecular‐genetic profile. Here, we review several diagnostic applications and future directions of these advanced neuroimaging techniques, including radiomics in preclinical models and patients with meningioma.

## Graphical abstract

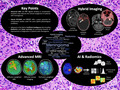

## 1 INTRODUCTION

The most frequently reported histology of all primary brain and other central nervous system tumors is meningioma and comprises 37.6% with an average annual incidence rate of 8.58 patients per 100,000 population [1].

Contrast‐enhanced structural magnetic resonance imaging (MRI) is routinely used in meningioma patients for defining the tumor extent, treatment planning, and follow‐up after treatment, especially for the diagnosis of tumor recurrence. Additionally, computed tomography (CT) allows, besides identifying calcifications for differential diagnosis, the diagnosis of osseous involvement of the adjacent skull bone [2, 3], which is of particular value for meningioma delineation and treatment‐decisions. While structural MRI is exceptional in providing information on both the central nervous system anatomy and meningiomas, advanced neuroimaging techniques offer the ability to yield additional information regarding tumor biology at both the functional and molecular levels. In neurooncology, these techniques are usually based on MRI, e.g., perfusion‐weighted imaging (PWI), diffusion‐weighted imaging (DWI), MR spectroscopy (MRS), and positron emission tomography (PET).

Moreover, artificial intelligence offers the potential to extract additional imaging features from routinely acquired MRI, CT, and advanced imaging techniques. Importantly, these features quantify image characteristics that are beyond human perception. In combination with clinical parameters or molecular markers, mathematical or machine learning models can be developed for an improved assessment of prognosis or the non‐invasive prediction of molecular‐genetic alterations. The development of these models based on quantitative features computed from medical images is called radiomics [4, 5, 6, 7] and allows linking imaging phenotypes to a tumor's molecular‐genetic profile, a field commonly referred to as radiogenomics. The latter is also of particular interest because efforts are currently ongoing to incorporate molecular profiling into the diagnostic work‐up to improve the characterization of meningiomas, e.g., in terms of prediction of the biological behavior [8].

Furthermore, deep learning‐based radiomics uses artificial neural networks that automatically extract high‐dimensional features from the images at different abstraction levels. As a result, characteristic image patterns are autonomously identified, learned, and used for classification [9].

Here, we review several diagnostic applications and future directions of these advanced neuroimaging techniques, including radiomics in preclinical models and patients with meningioma.

## 2 NEUROIMAGING OF PRECLINICAL MENINGIOMA MODELS

Various meningioma animal models were successfully established during the last years to study the mechanisms of tumor initiation and progression and the efficacy and toxicity of novel treatment approaches [10, 11]. Ideally, not only the presence and exact location of the tumor growth can be visualized, but also the rate of tumor growth can be quantified in longitudinal measurements before or after treatment. Thus, slowing down tumor growth or even tumor regression following treatment may become detectable in individual animals. An essential prerequisite for such longitudinal tumor imaging is a non‐invasive nature of the imaging modality that allows examinations of even weakened animals (e.g., due to treatment side effects). Currently, mice are mainly used as animal models, and therefore imaging modalities with a high spatial resolution are required to depict a meningioma as a total nude mouse brain has only a volume of about 450 mm^3^. Additionally, to detect pathological relevant changes in meningioma growth, imaging with submillimeter range resolution is crucial to evaluate putative cancer treatment effects.

There are basically two options to distinguish the meningioma from surrounding healthy tissue under in vivo conditions. First, already existing structural changes related to the tumor growth can be visualized. Second, tumor cells can be labeled either intrinsically (i.e., tumor cells express a detectable marker) or extrinsically (i.e., an external applied detectable marker specifically binds to tumor cells). Besides others, frequently used non‐invasive imaging modalities are bioluminescence imaging, MRI, and PET.

### 2.1 Bioluminescence imaging

The labeling of tumor cells with an easily detectable marker, such as different luciferases (i.e., firefly luciferase, marine Renilla luciferase, or Oplophorus luciferase), is an intriguing approach to identify the location and putative spreading of the meningioma over time in individual animals [12]. Because luciferase expression is cell‐specific, the bioluminescence signal indicates the presence and location of meningioma cells. An augmented signal intensity relates to an increased number of cells that express the respective marker and thus indicates tumor growth. Besides, bioluminescence provides information on tumor cell viability. Luciferases only produce light in the presence of the substrates luciferin or coelenterazine, which have to be applied before imaging, and of intracellular adenosine triphosphate, oxygen, and Mg^2+^. Thus, light is only generated in living cells [13, 14]. This also means that dead tumor cells, infiltrating host cells, and tumor cell debris do not contribute to the bioluminescence signal [15]. Thus, the total tumor size does not necessarily relate to the measured bioluminescence signal. Nevertheless, one should keep in mind that artificial expression of luciferase may itself affect the immune response toward these cells, which may eventually result in reduced growth of these reporter‐labeled cells [16, 17, 18].

So far, human immortal IOMM‐Lee cells (intraosseous malignant meningioma‐derived cell line) were transfected with firefly luciferase. As early as 3 days after implantation at the skull base, bioluminescence imaging successfully detected the tumor, and, subsequently, signals increased nearly exponentially until day 18 [19]. Although imaging was highly sensitive, the exact location and extent of the meningioma had to be verified by subsequent histological analysis. In a similar study, another human immortal cell line (CH‐157‐MN), as well as IOMM‐Lee cells, was implanted at the skull base or at the cerebral convexity of 3‐week‐old immunodeficient mice. Bioluminescence signals were measured biweekly starting 1 week after implantation [20]. According to the measured bioluminescence signals, almost logarithmic tumor growth was detected until day 17–18.

In addition to human immortal cell lines, mouse neonatal arachnoidal cells with inactivated *Nf2* and *Cdkn2ab* genes were injected at the craniocervical junction in immunocompetent 6‐week‐old mice. These cells, which were also co‐transfected with a luciferase reporter gene, developed to higher‐grade meningioma. In these mice, bioluminescence imaging could detect growing tumors at the skull base or convexity of 3‐month‐old mice [21].

In general, bioluminescence imaging provides an improved approximation of the actual tumor burden, but only limited information about the tumor's exact spatial distribution. Therefore, it has to be considered to transfect meningioma tumor cells with a secreted luciferase and, for tumor growth monitoring, a corresponding blood luciferase reporter gene assay is necessary [22]. This approach is an inexpensive, rapid, nevertheless it is a sensitive method to measure tumor growth and response to various treatments.

### 2.2 Magnetic resonance imaging

So far, MRI has the highest spatial resolution (up to 25 µm^2^ in‐plane) for soft tissue in vivo imaging. Therefore, several anatomical MRI studies were performed to delineate and quantify the volume of meningiomas. The first study was published in 2003 using a 1.5 T clinical MRI system. In that study, xenografts containing IOMM‐Lee cells were implanted at the skull base of mice. T1‐weighted MRI could detect the developing tumor after 14 days. These images already had an in‐plane resolution of about 100 × 100 µm, but a slice thickness of 1.5 mm hampered an accurate volume determination [23]. With the advent of ultra‐high field MRI animal systems, spatial resolution increased considerably. The use of 9.4 T animal scanners combined with cryo‐coils allows scanning with an in‐plane spatial resolution of 50 × 50 µm, and a slice thickness of 250 µm.

**FIGURE 1:**
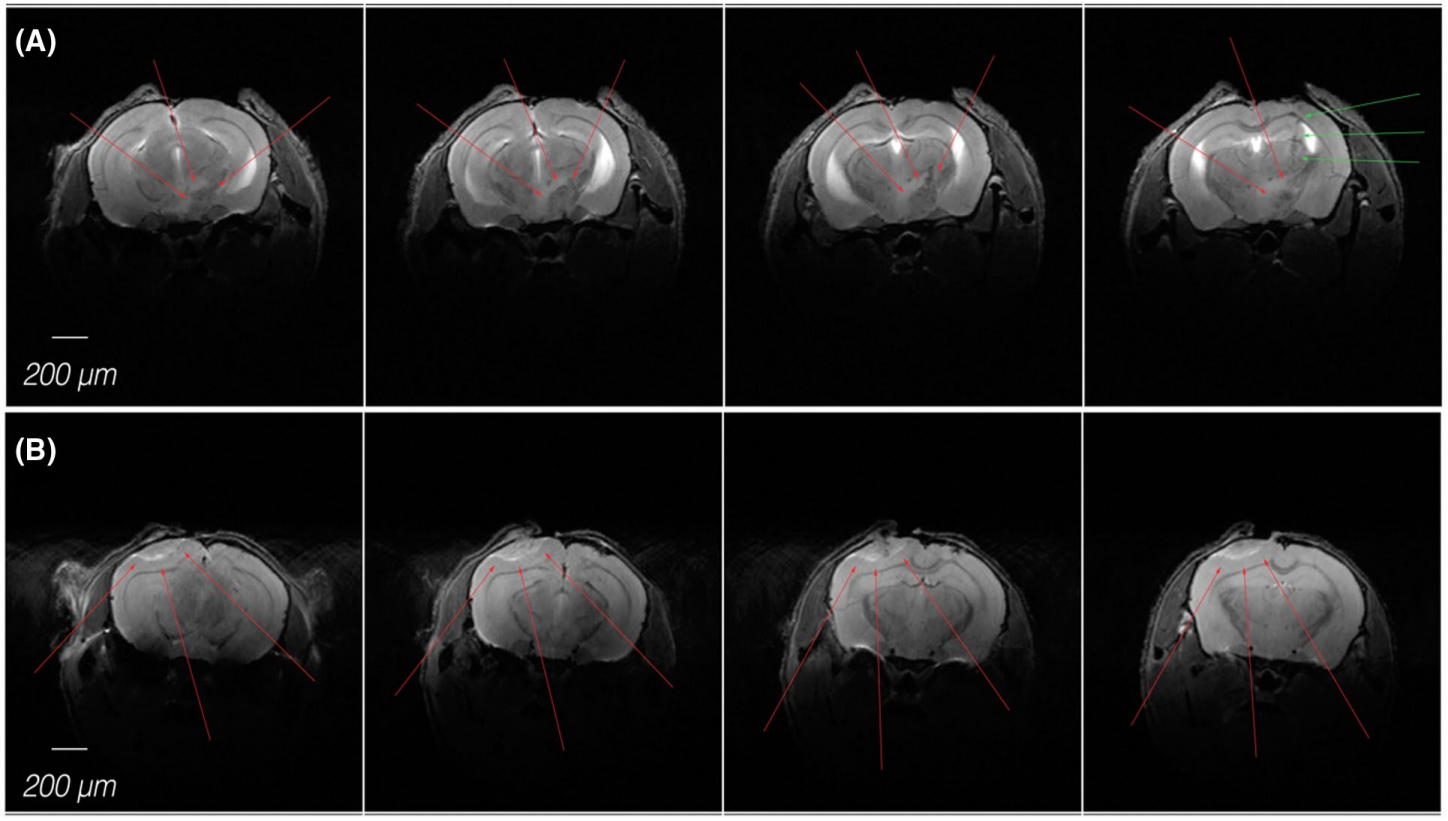
T2‐weighted anatomical MR images visualize the location and size of a meningioma either at skull base (A) or at the cerebral convexity (B) of the mouse brain. In‐plane resolution of 52 × 52 µm (field of view, 20 × 20 mm; imaging matrix, 384 × 384) allows for a clear separation of the meningioma (red arrows) from the surrounding tissue. Green arrows indicate the site for implanting the meningioma cells

In most cases, T2‐weighted images were sufficient to distinguish the meningioma from the surrounding normal brain tissue (Figure 1). Up to now, various xeno‐ and allografts were visualized and quantified by anatomical MRI. For example, the impact of micro‐RNA 145 on IOMM‐Lee cell growth at the cortical curvature was determined by T2‐weighted MRI [24]. Similarly, an inhibitory effect of temsirolimus, regorafenib, and sorafenib on IOMM‐Lee cells growth at the cortical curvature could be verified by serial T2‐weighted measurements [25, 26]. Furthermore, the growth of KLF4K40Q transfected IOMM‐Lee cells at the convexity and skull base could also be followed and quantified by T2‐weighted MRI [27]. A similar imaging approach was also used to follow the slowly growing KT21 meningioma cells at the convexity [28].

**FIGURE 2:**
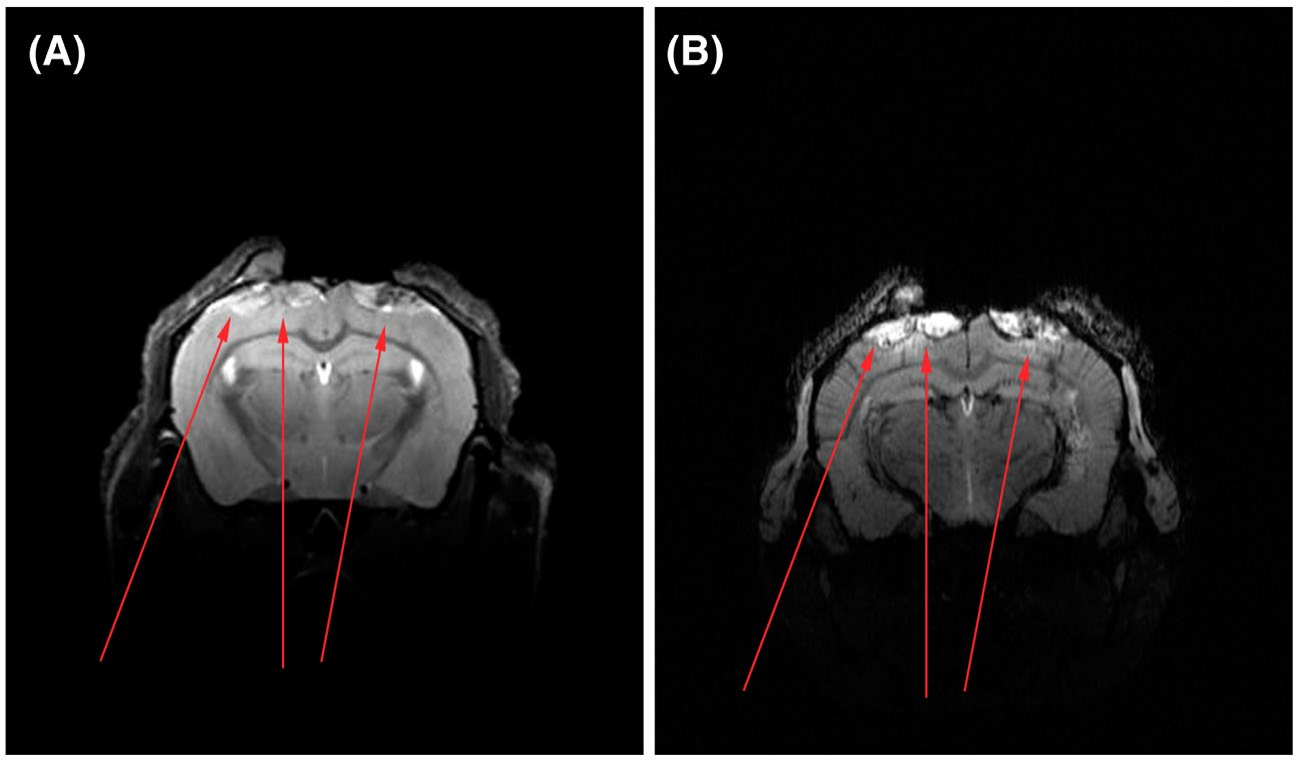
Meningiomas (red arrows) at the cerebral convexity of the mouse brain delineated using T2‐weighted MRI (A) and a subsequently obtained T1‐weighted MRI after intravenous application of a gadolinium‐based contrast agent (B)

In meningiomas, the contrast in T1‐weighted images is increased by the intravenous application of a gadolinium‐based contrast agent (Figure 2). Corresponding histology confirmed widespread vascularization and, often, hemorrhage in the tumor [29]. In combination with bioluminescence imaging at the craniocervical junction, contrast‐enhanced MRI visualized meningioma developed from mouse neonatal arachnoidal cells with an inactivated *Nf2* as well as *Cdkn2ab* gene [21]. Besides detecting xeno‐ or allografts, contrast‐enhanced T1‐weighted MRI allows exposing abnormal meningeal proliferations in mice deficient in *Nf2* and *p16* [30]. These spontaneously developing meningiomas were subsequently verified by histology.

**FIGURE 3:**
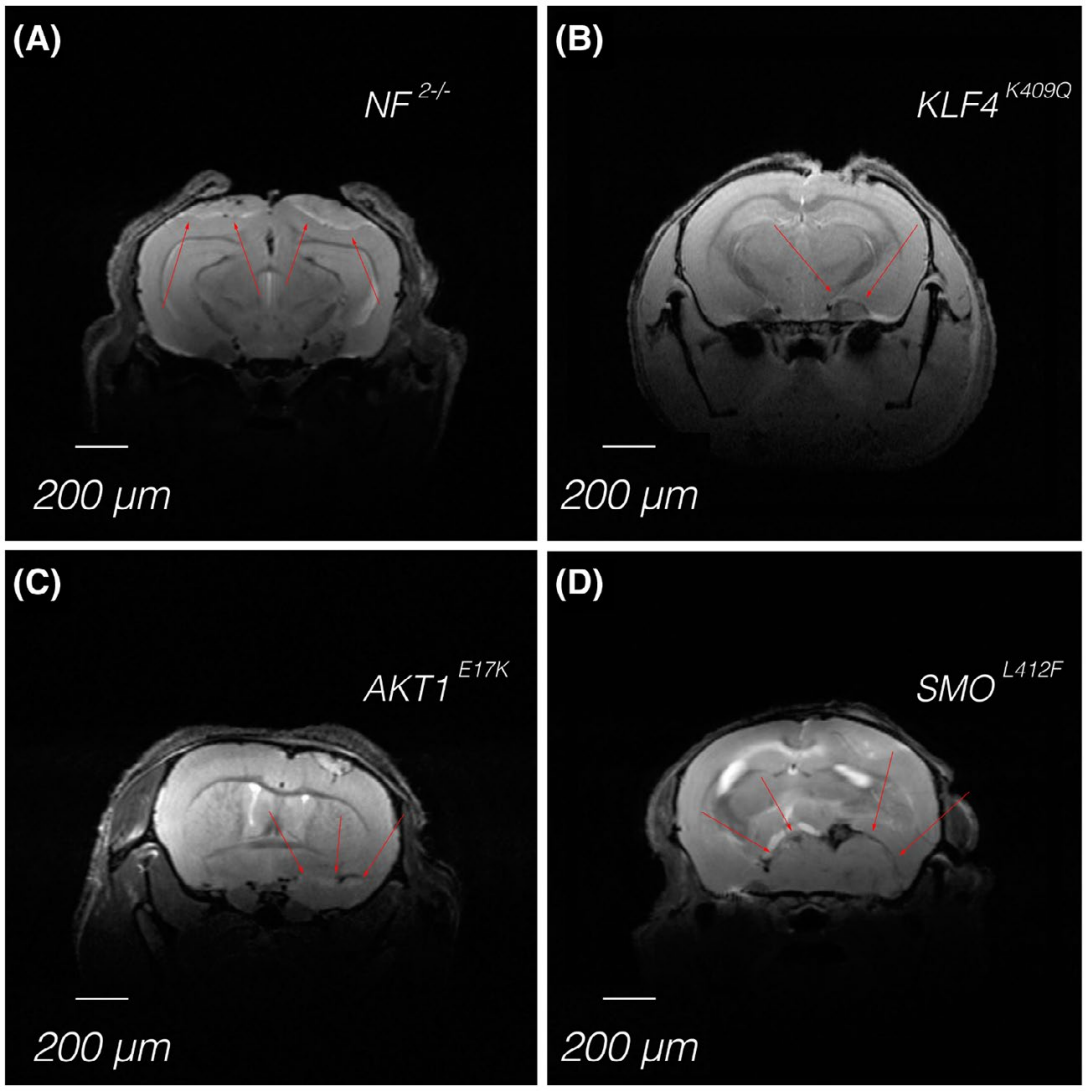
High‐resolution MR imaging of meningioma in Swiss nude mice derived from different cell lines carrying either deletion of *NF2* (A) or a mutation in *AKT1* (B), *KLF4* (C), or *SMO* (D). Red arrows indicate the location of the meningiomas

In summary, high‐resolution MRI is well suited to localize and quantify meningiomas that develop from various cell lines (Figure 3). The main advantage of high‐resolution MRI is the possibility to precisely delineate the tumor and visualize possible invasions in bone or even perforations through the skull base. Notably, a combination of different modalities may also increase the informative value. All imaging methods mentioned here can be used for longitudinal studies. In parallel, they can be used to localize and quantify the tumor size, e.g., MRI, or verify tumor cell viability, e.g., bioluminescence imaging. On the other hand, infrastructure and running costs for MRI are more extensive and expensive than bioluminescence imaging. Nevertheless, MRI offers the possibility to perform different imaging sequences (e.g., T1 with or without contrast agent, T2) during one imaging session. Furthermore, current developments in MRI nanoimaging agents, which are highly versatile for on‐demand covalent conjugation of various moieties, including proteins [31], may further increase the contrast for meningioma in MR images.

### 2.3 Positron emission tomography

Meningioma cells are known to highly express somatostatin receptors (SSTR), predominantly the SSTR subtype 2 [32]. Consequently, somatostatin receptor ligands, such as ^68^Ga‐DOTA‐Tyr3‐octreotide (DOTATOC), ^68^Ga‐DOTA‐l‐Nal3‐octreotide (DOTANOC), or ^68^Ga‐DOTA‐D‐Phe1‐Tyr3‐octreotate (DOTATATE) that have high affinity to the SSTR2, were labeled with the positron‐emitting nuclide ^68^Ga and used to define the meningioma extent, particularly for treatment planning in patients with meningioma [33].

Currently, the number of animal studies in mice that use SSTR PET ligands is small. Soto‐Montenegro and colleagues evaluated a subcutaneous human meningioma CH‐157MN xenograft using the latter mentioned ^68^Ga‐labeled SSTR analogs. Of these, ^68^Ga‐DOTATATE had the best tumor‐to‐muscle uptake ratio, indicating that this tracer seems to be the best option for detecting meningiomas [34].

A further DOTATATE PET study evaluated subcutaneously implanted human CH‐157MN meningioma xenografts serially after inoculation. On day 20, the DOTATATE PET scan revealed a reduced tumoral tracer binding compared with earlier scans at days 7 and 13, assuming that this reflects necrotic areas within the tumor [35]. Although it has undisputable potential for research applications, PET studies using SSTR ligands have limitations, particularly for mouse imaging. First, the spatial resolution of preclinical PET is inherently limited by physical principles and usually in the range of 0.7–1 mm. Second, signal detection in tiny regions can be easily contaminated by surrounding regions, hampering tracer binding quantification. Third, the use of SSTR PET to quantify tumor growth over time requires a stable expression of somatostatin receptors during early and late stages.

## 3 USE OF ADVANCED MRI AND PET IN PATIENTS WITH MENINGIOMA

Contrast‐enhanced anatomical MRI is exceptional in providing detailed structural information of the central nervous system anatomy and brain neoplasms, although its specificity is comparatively poor [36, 37, 38, 39, 40]. Advanced MR techniques, including PWI techniques such as dynamic contrast‐enhanced (DCE) or dynamic susceptibility contrast (DSC) PWI and arterial spin labeling (ASL), apparent diffusion coefficients (ADC) obtained by DWI, and proton MRS [41, 42, 43], yield additional information regarding tumor biology, especially at the molecular, physiological, and functional levels.

PWI is a non‐invasive MRI technique to measure blood flow quantitatively. In Neuro‐Oncology, the parameter relative cerebral blood volume is frequently assessed. Most commonly, a gadolinium‐based contrast agent is used to assess tissue perfusion. DSC MRI uses the passage of the contrast agent to cause local magnetic field distortion (susceptibility effect) in the vicinity of the vessels resulting in a signal drop in T2‐ or T2*‐weighted MRI. DCE MRI is based on shortening of the T1‐relaxation time causing a signal increase in T1‐weighted MRI. ASL is another PWI method which does not require a contrast agent. Here, endogenous water molecules in blood vessels are magnetically labeled by applying a specific radiofrequency pulse. Passage of these labeled molecules through the tissue of interest leads to a reduction of signal intensity in proportion to the perfusion.

DWI is based on the measurement of Brownian motion of water molecules to generate an image contrast. DWI contrast uses two opposing gradient pulses; the first one induces a phase shift in water molecules, leading to a signal reduction. Subsequently, a second opposed gradient pulse is applied, which rephases the water molecules in the region of interest, leading to a recovery of the water signal.

Proton MRS is a non‐invasive method to detect selected water‐soluble metabolites in vivo. By the application of external magnetic fields, every metabolite has its characteristic magnetic field signature resulting in slightly different resonance frequencies with differential signals. These signal differences are used in MRS to identify the metabolites of interest.

## 4 MOST RELEVANT CLINICAL APPLICATIONS FOR ADVANCED MRI

### 4.1 Differential diagnosis

**FIGURE 4:**
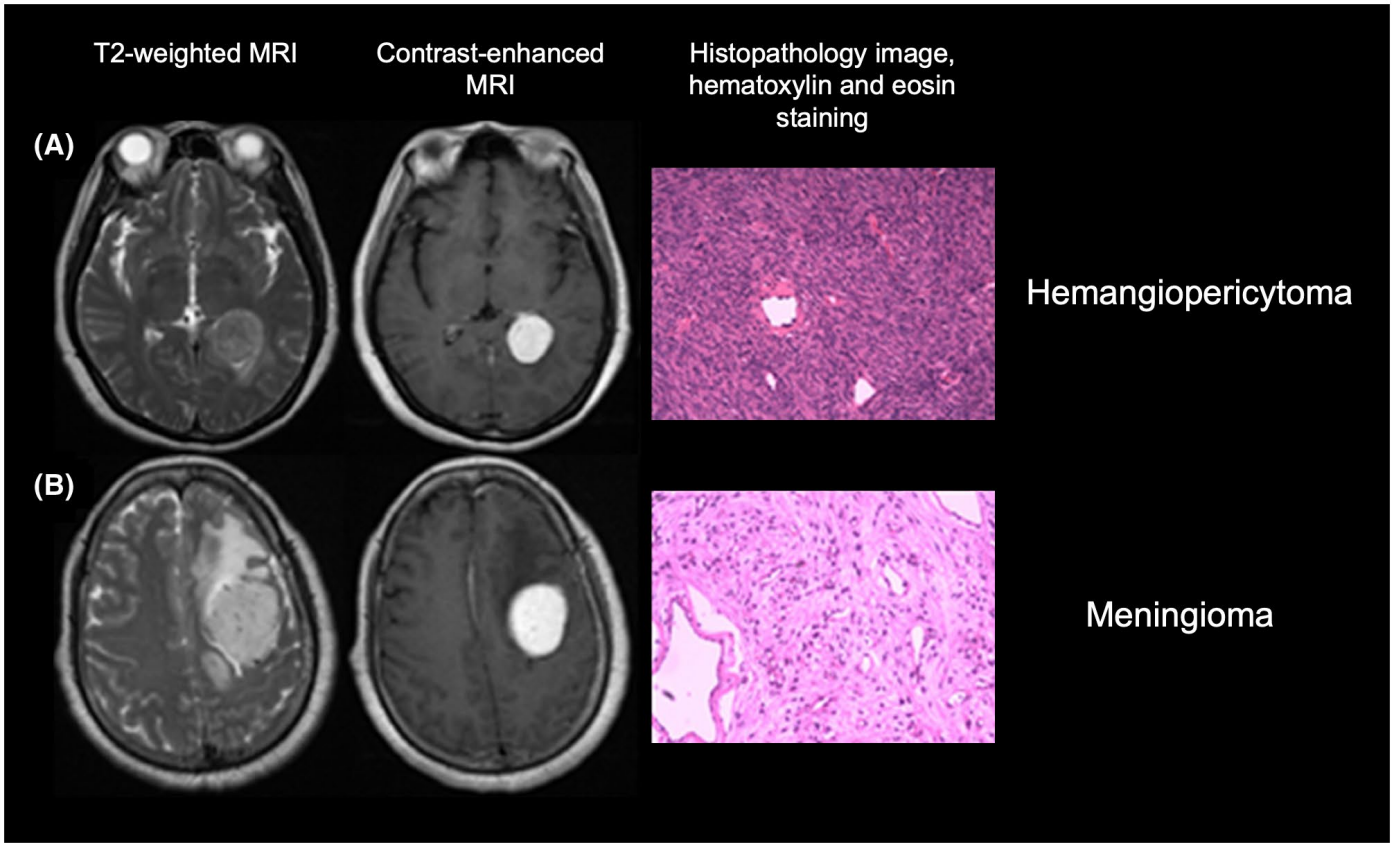
MRI scans and corresponding histopathological images with hematoxylin and eosin staining of two patients diagnosed with a hemangiopericytoma (A) and a meningioma (B). The similar appearance on conventional MRI makes a reliable differential diagnosis difficult. Here, the preoperative extraction of quantitative image features using radiomics provided additional diagnostic information to improve differential diagnosis. Modified from Wei et al. [103], under the terms of the Creative Common Attributions License (CC‐BY, version 4.0)

A wide variety of neoplastic and inflammatory diseases have a propensity for the dura mater or subdural space's involvement and may mimic meningioma. For example, dural‐based brain metastases, lymphomas, tumors of the solitary fibrous tumor spectrum (previously referred to as hemangiopericytomas) (Figure 4), as well as sarcoidosis and tuberculosis, may exhibit a meningioma‐like appearance [44, 45]. Furthermore, depending on the meningioma size, the distinction between an intraaxial and extraaxial origin may be difficult [46].

Perfusion MRI may differentiate between meningioma and dural metastases from different entities, especially breast cancer and colorectal carcinoma [47]. The cerebral blood volume in these brain metastases entities seems to be significantly lower than in meningiomas. By contrast, brain metastases from renal cell carcinoma or melanoma may also have elevated blood volumes, thereby hampering differential diagnosis of meningioma [48, 49, 50]. Using proton MRS, metabolic profile characterization may add valuable information for the differentiation between meningiomas and brain metastases [51, 52]. A considerable number of meningiomas exhibit a relatively high choline peak at 3.2 parts per million and an inverted doublet alanin peak centered at 1.45 parts per million [52, 53].

### 4.2 Meningioma grading

In patients with newly diagnosed meningioma, various imaging features derived from preoperative anatomical MRI (e.g., heterogenous contrast enhancement, perifocal edema, presence of a brain–tumor interface) may be associated with an atypical meningioma of the WHO grade II or a WHO grade III anaplastic meningioma [54]. Furthermore, perfusion MRI metrics seem to be of value to differentiate between WHO grade I and grade II or III meningioma. A study suggested that the cerebral blood volume accurately reflects vascular endothelial growth receptor expression and tumor grade in meningiomas and helps identify patients with WHO grade II or III meningioma [55]. Another study observed that perfusion patterns in cerebral blood flow maps derived from ASL are also of value for meningioma grading [56]. In that study, a heterogenous hyperperfusion or a lack of hyperperfusion was significantly associated with the presence of a high‐grade meningioma (i.e., WHO grade II or III).

A multicenter study included around 400 meningioma patients and suggested that also apparent diffusion coefficients derived from DWI can differentiate WHO grade I meningioma from grade II and III tumors with an accuracy of 73% [57]. Additionally, in that study, the proliferation marker Ki‐67 was significantly correlated with ADC derived from DWI. However, it has to be pointed out that predominantly older diffusion and perfusion MRI studies reported no additional value or discrepant results regarding meningioma grading [58, 59, 60].

### 4.3 Meningioma relapse risk stratification

For patient management and treatment decisions, the prediction of an early meningioma relapse, i.e., identifying meningioma patients with increased relapse risk is of great clinical importance. Notably, an earlier diagnosis of meningioma relapse using conventional MR or CT imaging may be impeded by specific tumor locations (e.g., skull base).

A retrospective study in 144 postoperative meningioma patients showed that DWI‐derived ADC provides additional information to predict an increased risk for meningioma relapse [61]. Besides other factors, patients with incomplete resection and low ADC had a significantly higher risk of progression or recurrence and may benefit from a more aggressive treatment strategy. Another study investigated the value of ex vivo ultra‐high‐field proton MRS at 11.4 T of resected tumor tissue to predict aggressive biological behavior in 64 WHO grade I‐III meningioma. The absolute concentrations of alanine and creatine, as well as the choline/glutamate and glycine/alanine ratios, were associated with an increased probability of rapid meningioma relapse [62]. By contrast, in vivo MRS has both limited spectral resolution and precision, thereby hampering equivalent analyses, especially of alanine and glutamate.

## 5 MOST RELEVANT CLINICAL APPLICATIONS FOR PET IMAGING

Several tracers addressing different molecular structures or pathophysiological pathways in meningioma cells are available [33]. Because of the overexpression of SSTRs in meningiomas [32, 63, 64], radiolabeled SSTR ligands are particularly used to visualize meningioma tissue. The SSTR subtype 2 is the most abundant isoform with almost 100% expression in meningiomas [32]. The most commonly applied SSTR ligands for PET imaging in patients with meningioma are DOTATOC and DOTATATE. After labeling with ^68^Ga, these ligands are frequently used as tracers for imaging of neuroendocrine tumors, which likewise express high levels of SSTR [65]. ^68^Ga has a physical half‐life of 68 minutes and can be produced with a ^68^Ge/^68^Ga generator, enabling in‐house production without an on‐site cyclotron. PET ligands to SSTR provide high sensitivity with excellent target‐to‐background contrast due to low uptake in bone and healthy brain tissue [66, 67]. Currently, the number of PET examinations in meningioma patients is steadily increasing.

The l‐amino acid transporter system mediates the uptake of radiolabeled amino acids such as [^11^C‐methyl]‐l‐methionine (MET) and O‐(2‐[^18^F]‐fluoroethyl)‐l‐tyrosine (FET). Increased uptake is seen in slow‐growing tumors such as meningiomas [68, 69].

### 5.1 Meningioma detection

Due to a meningioma localization with low contrast on MRI or CT, e.g., at the skull base with or without osseous involvement of the adjacent skull bone, tumor detection and delineation may be complicated if standard anatomical cross‐sectional imaging techniques are applied. Furthermore, meningiomas can be obscured by calcifications or MRI artifacts. The recent body of literature has suggested that SSTR PET adds valuable diagnostic information to MRI or CT to overcome these issues.

**FIGURE 5:**
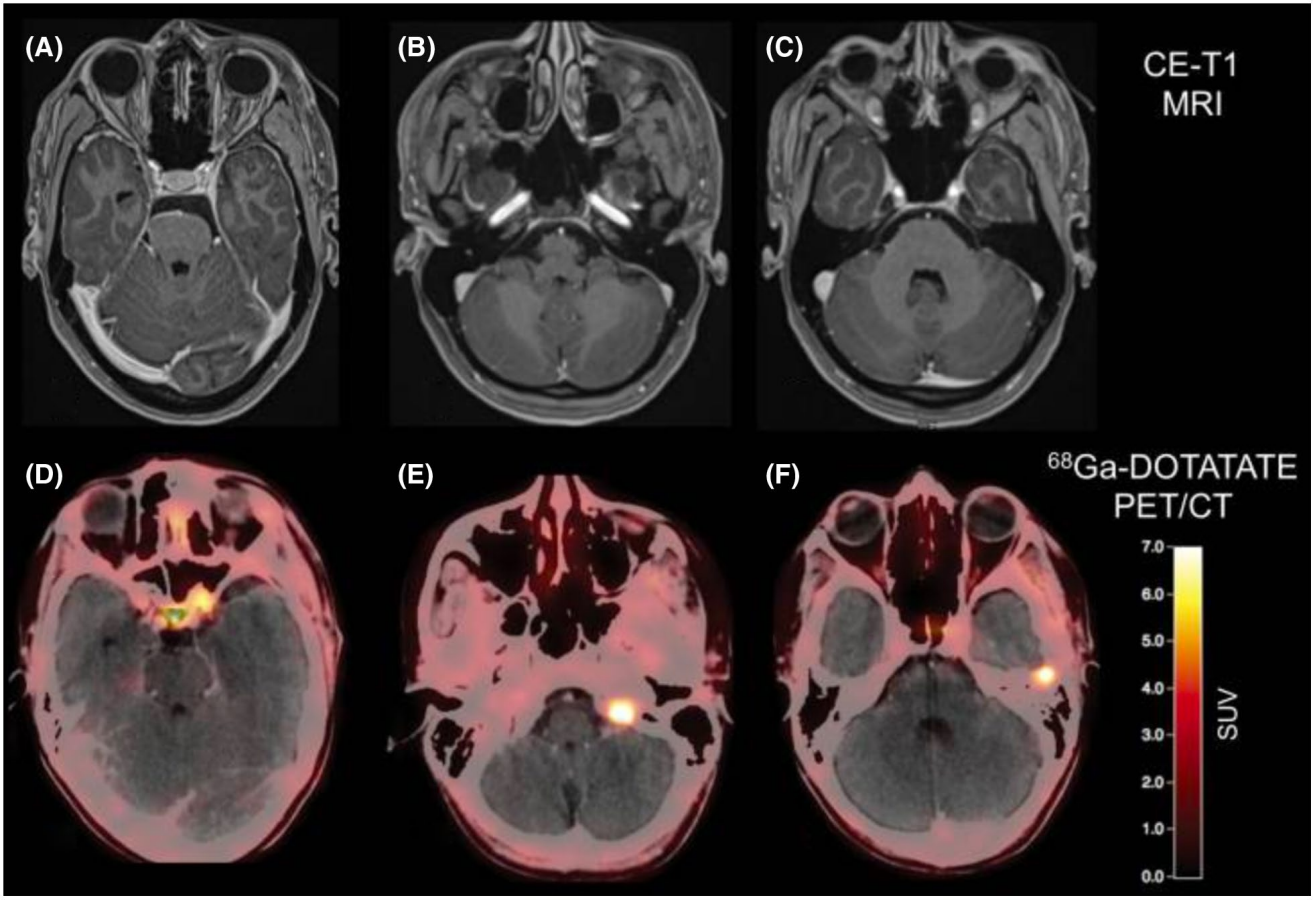
Postoperative contrast‐enhanced MRI and DOTATATE PET/CT of a patient after resection of a WHO grade I meningioma show residual tumor located at the left internal carotid artery and a tumor at the tip of the left orbit (A and D). Surprisingly, two additional meningiomas were also visible on the DOTATATE PET/CT (E and F), without corresponding contrast enhancement on MRI (B and C) (reproduced from Galldiks et al. [33], with permission from Oxford University Press)

A study compared contrast‐enhanced MRI with DOTATOC PET in 190 meningioma patients before radiotherapy and reported that all meningiomas were detected by PET, whereas contrast‐enhanced MRI detected only 90% of these meningiomas. These findings indicate that the improved sensitivity for DOTATOC PET may identify additional meningiomas even if MRI is negative [66] (Figure 5).

DOTATATE PET studies with histological validation of imaging findings revealed a more precise tumor extent delineation than contrast‐enhanced MRI [67, 70]. Furthermore, in meningiomas with complex growth patterns, i.e., involvement of the sagittal or cavernous sinus, the orbita, or infiltration of other osseous structures, PET using DOTATATE and DOTATOC was also reported to provide an improved tumor delineation compared with MRI [71, 72, 73]. Another study suggested that DOTATATE PET helps discriminate optic nerve sheath meningiomas from other lesions affecting the optic nerve [74]. Similarly, studies using amino acid PET reported an improved meningioma delineation compared with MRI [68, 75, 76].

Moreover, PET images can be integrated into MR‐based neuronavigation systems for image‐guided neurosurgery, and the additional information in terms of tumor extent can be used for the intraoperative guidance of resection, e.g., in complex skull base meningiomas.

### 5.2 Meningioma grading

The uptake of radiolabeled glucose (2‐[^18^F]‐fluoro‐2‐deoxy‐d‐glucose; FDG) correlates significantly with the WHO grade in meningiomas [77, 78], but as a significant limitation, its uptake is not specific for neoplastic tissue and may be increased in inflammatory processes [79]. Regarding PET ligands to SSTR, DOTATATE binding significantly correlates with tumor growth rates in WHO grade I and II meningiomas but is abolished in anaplastic meningiomas [80]. Data on the amino acid tracer MET labeled with ^11^C suggest a correlation with proliferative activity in patients with meningioma [81], but are controversial for non‐invasive meningioma grading [82, 83]. Furthermore, due to the short half‐life of ^11^C of 20 minutes, its use is strictly limited to centers with an on‐site cyclotron. Preliminary findings revealed that static and dynamic FET parameters might provide additional information for non‐invasive grading of meningiomas [69].

### 5.3 Radiotherapy planning

Target definition plays a crucial role in the planning of high precision radiotherapy using fractionated radiotherapy or radiosurgery. Despite using the bone window on CT scans, it is challenging to define the infiltration depth in meningiomas with transosseous growth. In these cases, PET imaging may prove helpful. For example, a DOTATATE PET study focusing on transosseous growing meningiomas showed a higher specificity than standard MRI (100% vs. 83%) [70].

Using SSTR ligands, an optimized target volume delineation for fractionated radiation therapy in WHO grade I‐III meningiomas could be obtained using DOTATOC PET co‐registered to CT and MRI [72]. In all patients, DOTATOC PET provided additional information on the meningioma extent for fractionated stereotactic radiotherapy planning. These results were confirmed by subsequent studies [73, 84, 85, 86].

Furthermore, amino acid PET can also be integrated into radiation treatment planning [87] and significantly influence target volume definition in meningioma patients. Astner and colleagues demonstrated that in the vast majority of patients with skull base meningiomas treated with fractionated radiotherapy, MET PET addition changed the target volumes considerably [68]. In that study, MET PET detected additional tumor areas, which were not visualized on conventional CT or MRI, leading to a target volume enlargement of almost 10%. Furthermore, areas without tumor affection could be excluded from the radiation field, and eloquent structures, such as the optic nerves, the chiasm, or the pituitary gland, could be spared more effectively [68]. Subsequently, it has been demonstrated that the addition of amino acid PET to CT and MRI helps significantly lower the interobserver variability than either modality alone [75, 76].

### 5.4 PET during the follow‐up of meningioma patients

The recent literature has suggested that SSTR PET can also help differentiate meningioma relapse from post‐therapeutic reactive changes, including radiotherapy [45, 66, 67, 88]. For example, Rachinger and colleagues reported a higher specificity for DOTATATE PET compared with standard MRI (74% vs. 65%) [67].

A recent study has suggested that the intraoperative estimation of meningioma extent for resection using Simpson grades is inferior compared with DOTATATE PET [89]. Although 62.5% of patients had a meningioma resection extent according to the Simpson grade I or II, DOTATATE PET revealed tumor remnants [89].

The initial case series also reported that SSTR PET seems to be valuable to detect extracranial metastatic meningioma involving the liver, lung, and bone [90, 91, 92].

## 6 OTHER IMAGING MODALITIES

### 6.1 Optical imaging

Besides other techniques, Raman spectroscopy is a powerful optical imaging method which allows to analyze the biochemical composition of tissue to differentiate neoplastic from normal tissue. By shining monochromatic laser light onto a sample obtained from brain surgery, this technique detects scattered light to measure the vibrational energy modes of a sample. A small amount of the scattered light shifts in energy from the laser frequency because of interactions between the incident electromagnetic waves and the vibrational energy levels of the molecules in the sample. Plotting the intensity of the shifted light against the frequency creates a Raman spectrum of the sample.

Initial studies suggest that Raman spectroscopy has a high diagnostic accuracy to differentiate between glioma subtypes, brain metastases, and meningioma [93, 94]. Another study highlighted the clinical potential of this technique for the determination of the meningioma grade, i.e., the differentiation between WHO grade I and II [95].

### 6.2 Intraoperative ultrasound including elastography

For neurosurgical interventions, intraoperative imaging guidance is fundamental to achieve a complete tumor resection and to preserve neurological functions. In this regard, intraoperative ultrasound is a reliable method to obtain real‐time information during brain surgery. Furthermore, the biomechanical properties of tissues correlated to histology, and neuropathological findings have also received increased attention in recent years. Ultrasonographic elastography imaging is able to evaluate intraoperatively the elastic properties of tissues such as tissue hardness to distinguish pathologic and healthy areas. An increasing body of literature suggests that elastographic ultrasound patterns may help to identify different brain tumor types, i.e., gliomas, metastases, and meningiomas [96, 97].

## 7 ARTIFICIAL INTELLIGENCE: RADIOMICS AND RADIOGENOMICS IN PATIENTS WITH MENINGIOMA

**FIGURE 6:**
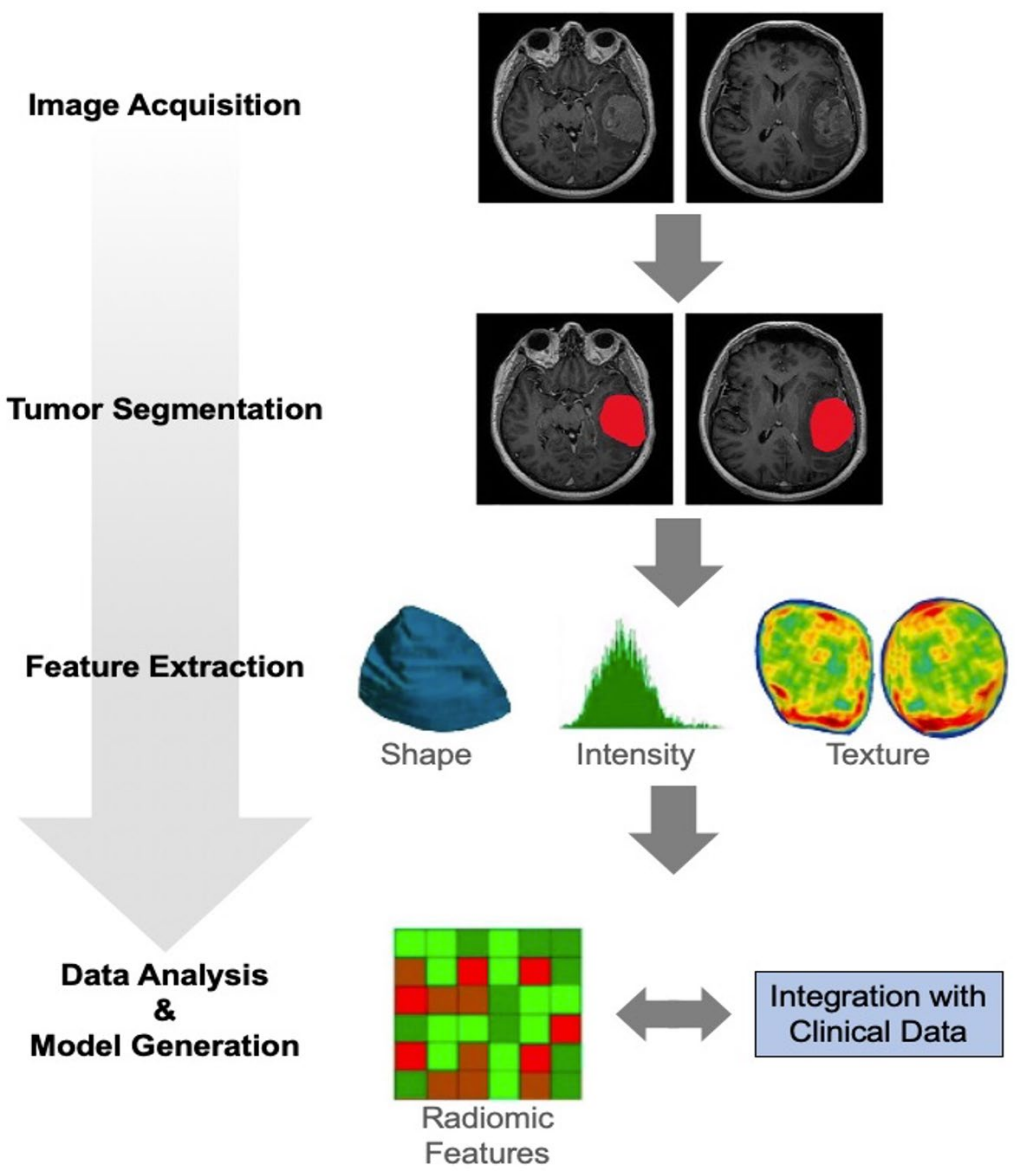
Schematic representation of the radiomics workflow. Following image acquisition, volumes‐of‐interest within tumor subregions (e.g., contrast enhancement, T2/FLAIR signal hyperintensity) are manually or automatically segmented. Most frequently, shape, intensity, and textural features are calculated. Subsequently, radiomics features are combined with clinical data (e.g., survival times, neuropathology findings), and a mathematical model related to the research question can be generated. Adapted from Gu et al. [130], under the terms of the Creative Common Attributions License (CC‐BY, version 4.0)

A subdiscipline of artificial intelligence dealing with the computation, identification, and extraction of image features for the generation of mathematical models related to the research purpose (e.g., to improve diagnostics) is termed radiomics. Radiomics is usually applied to routinely acquired imaging modalities, thereby allowing additional data analysis at a low cost. Since radiomics features are either mathematically predefined (feature‐based radiomics) or generated from the data by training computational models (deep learning‐based radiomics), the results are more robust, reliable, and reproducible. Radiogenomics, a subdiscipline of radiomics, aims to correlate radiomics features with molecular markers, genetic mutations, or chromosomal aberrations. Figure 6 shows a representative feature‐based radiomics workflow.

### 7.1 Differentiation between meningiomas and other brain tumors

**FIGURE 7:**
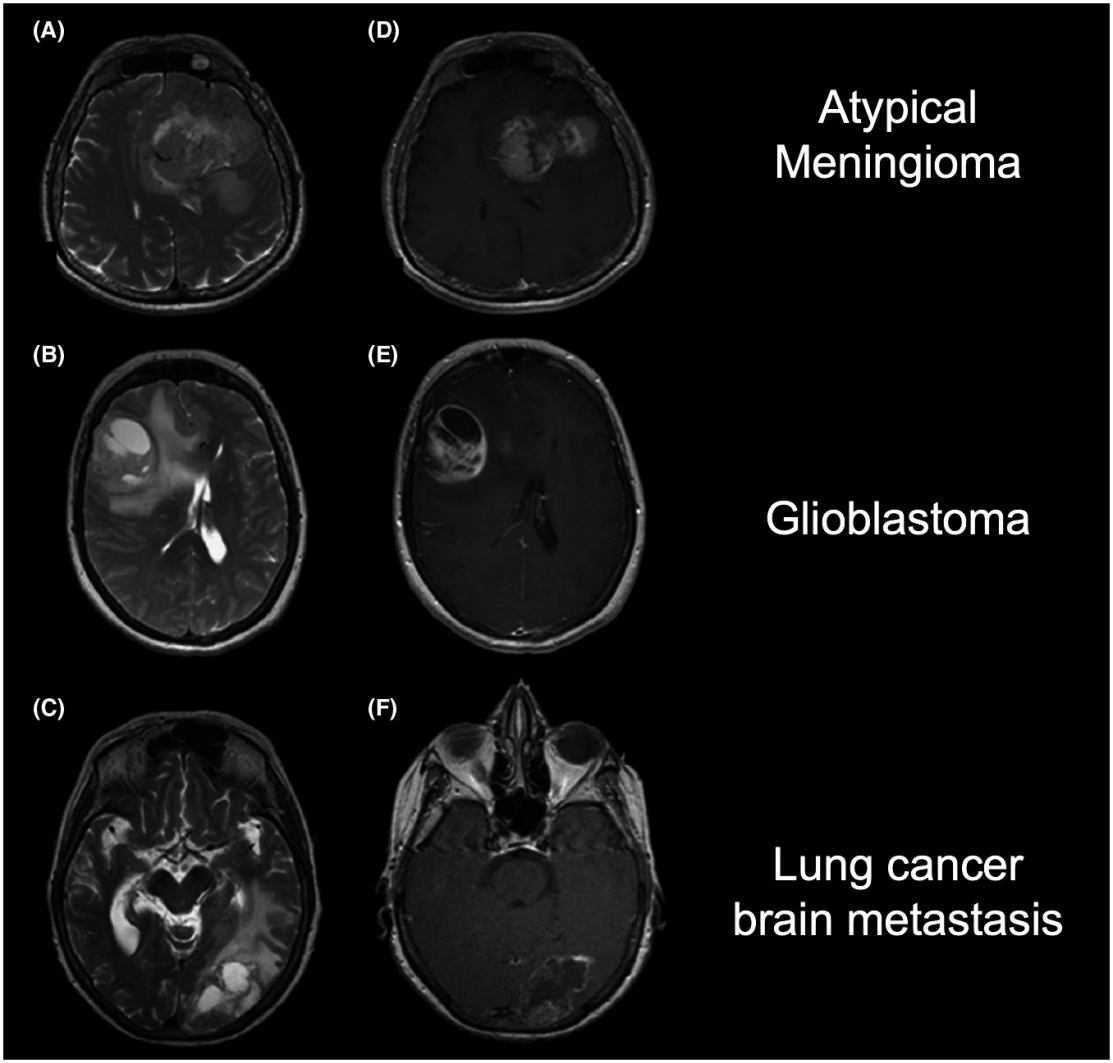
Representative T2‐weighted (A–C) and contrast‐enhanced T1‐weighted MR images (D‐F) of an atypical meningioma (top row), a glioblastoma (middle row), and a lung cancer brain metastasis (bottom row). Notably, the similar radiological findings make it difficult to differentiate between these three tumor entities. Here, advanced neuroimaging techniques may provide additional diagnostic information to improve differential diagnosis. Adapted from Svolos et al. [101], with permission from Elsevier

The differentiation between different brain tumor types based on conventional MRI alone is challenging due to similar imaging findings such as contrast enhancement and perifocal edema (Figure 7). Therefore, artificial intelligence and machine learning methods have been used to differentiate meningiomas from other brain tumor types. The differentiation between meningiomas, gliomas, and tumors of the pituitary gland using modified local binary pattern feature extraction methods was investigated by Kaplan and colleagues [98]. Local binary patterns describe the texture pattern in neuroimages and reflect the correlation among pixels within a local area [99]. In that study, the dataset consisted of more than 3000 T1‐weighted MRI slices from 233 patients. The identified local binary patterns differentiated between meningiomas, gliomas, and pituitary tumors with an accuracy of 96%.

In addition to structural MRI, Shrot and co‐workers included perfusion‐ and diffusion‐weighted MRI from 141 patients differentiate between meningioma, glioblastoma, primary central nervous system lymphoma, and brain metastases [100]. The final support vector machine classifier yielded more than 90% classification accuracy for all investigated tumor types. These results are in line with results reported in an earlier study using a similar methodology [101].

Li et al. aimed at a preoperative distinction of malignant hemangiopericytoma from angiomatous meningioma based on structural MRI and DWI [102]. Clinical and textural features were generated, and the performance of the radiomics model was compared with the rating of three experienced neuroradiologists. A support vector machine classifier based on T1‐weighted MRI revealed the best diagnostic performance with an area under the curve (AUC) of 0.90, outperforming the neuroradiologists' rating (AUC, 0.73). Similar results could be confirmed by a subsequent study [103].

### 7.2 Identification of meningioma subtypes

In clinical routine, the mainstay of brain tumor classification, including meningiomas, performed by neuropathologists is based on morphological criteria. Notably, various meningioma subtypes exhibit only minor morphological variations and may challenge meningioma subtyping. To facilitate the neuropathological diagnosis of meningothelial, fibroblastic, transitional, or psammomatous WHO grade I meningioma, Fatima and colleagues [104] developed a hybrid classification technique based on radiomics features of these four subtypes. The selected features were used to train a neural network classifier and yielded an average accuracy of more than 90%.

### 7.3 Meningioma grading and outcome prediction

For meningioma grading using radiomics features based on structural MRI, radiomics models achieved diagnostic accuracies between 76% and 93% for the differentiation of WHO grade I from WHO grade II or III meningiomas [105, 106, 107, 108, 109]. The accuracy could be further increased to 97% by integrating advanced MRI techniques such as DWI to radiomics models [110, 111, 112, 113, 114, 115, 116]. Similarly, several studies developed deep learning‐based radiomics models based on structural MRI for meningioma grading and achieved diagnostic accuracies between 80 and 83% [117, 118, 119]. In addition, Banzato and colleagues [120] developed a deep learning model based on ADC maps derived from DWI that provided a relatively high diagnostic accuracy of 94% for meningioma grading.

Importantly, a recent study has evaluated the robustness of radiomics based on structural MRI data from 25 different scanners acquired using 126 different imaging protocols [121]. Despite that heterogeneity, the developed deep learning radiomics model yielded a diagnostic accuracy of 74% for meningioma grading, indicating high robustness.

The 2016 edition of the WHO Classification of Tumors of the Central Nervous System has introduced the criterion of brain invasion to diagnose meningiomas of the WHO grade II [122]. Brain invasion is associated with a higher rate of tumor relapse and unfavorable prognosis [123, 124, 125, 126]. Consequently, several studies investigated the potential of radiomics for the non‐invasive identification of brain invasion [127, 128, 129]. For example, in more than 450 patients, Joo and co‐workers evaluated structural MRI radiomics features calculated from peritumoral edema and brain‐to‐tumor interface [127]. The final model combining the best six radiomics features and peritumoral edema volume yielded an AUC of 0.91 to identify brain invasion.

Initial studies suggest that prognostic models based on clinical parameters and radiologic and radiomic features may preoperatively identify meningiomas at risk for poor outcomes. For example, Morin et al. used preoperative structural and diffusion‐weighted MRI scans from 303 patients who underwent resection of 314 meningiomas (57% WHO grade I, 35% grade II, and 8% grade III) to extract 16 radiologic and 172 radiomic features [115]. The colleagues observed that both radiologic and radiomic predictors of adverse meningioma outcomes were significantly associated with molecular markers of aggressive meningioma biology, such as somatic mutation burden, DNA methylation status, and *FOXM1* expression. Furthermore, multivariate analyses revealed that radiomics features obtained from diffusion‐weighted MRI were significantly associated with WHO meningioma grades, local failure, and overall survival.

## 8 CONCLUSIONS AND OUTLOOK

Advanced MRI techniques and PET ligands binding to SSTR can improve the clinical management of patients with meningioma. The translation of these imaging modalities is also of great interest in the light of emerging high‐throughput methods such as radiomics. Furthermore, the increasing use of hybrid PET/MRI systems offers an immense research potential for comparative studies under the same (patho‐) physiological conditions. Besides, the increasing availability of ultra‐high field MRI scanners with higher spatial resolution may help develop novel MRI methods in meningiomas because almost all MRI contrasts benefit from the improved signal‐to‐noise ratio. Nevertheless, the implementation of advanced MRI and PET methods in clinical routine requires the validation of neuroimaging findings by neuropathology.

Furthermore, various radiomics approaches are promising in terms of the improvement of diagnostics in patients with meningioma. Importantly, the use of advanced imaging techniques may even further improve radiomics models. This may accelerate the translation of decision support systems based on artificial intelligence into daily clinical practice.

## CONFLICT OF INTEREST

Related to the present work, all authors report no conflicts of interest.

## AUTHOR CONTRIBUTIONS

Norbert Galldiks and Philipp Lohmann conceptualized the review article. A review of the literature was performed by Norbert Galldiks, Frank Angenstein, and Philipp Lohmann. The first draft of the manuscript was written by Norbert Galldiks, Frank Angenstein, and Philipp Lohmann. All other authors commented on previous versions of the manuscript. All authors read and approved the final manuscript.

## Data Availability

Data sharing is not applicable to this article as no new data were created or analyzed in this study.
